# Vibronic and Excitonic Structure of a Template-Engineered Molecular-Graphene Heterostructure

**DOI:** 10.1021/acs.nanolett.6c01284

**Published:** 2026-07-21

**Authors:** J. Kunc, B. Morzhuk, V. Stará, D. Varshney, M. Shestopalov, K. Matějka, M. Rejhon, J. Novák, J. Čechal

**Affiliations:** † Faculty of Mathematics and Physics, Institute of Physics, 37740 Charles University, Ke Karlovu 5, Prague 2 CZ-12116, Czech Republic; ‡ Central European Institute of Technology (CEITEC), 48274 Brno University of Technology, Purkyňova 123, Brno CZ-61200, Czech Republic; § Department of Condensed Matter Physics, 37748 Masaryk University, Kotlářská 267/2, Brno CZ-61137, Czech Republic

**Keywords:** molecular-graphene heterostructure, electron−phonon coupling, Holstein Hamiltonian, 2,3,6,7,10,11-hexamethoxytriphenylene

## Abstract

Strong electron–phonon coupling described by the Holstein Hamiltonian governs the optical response of many molecular quantum materials. Here, we investigate epitaxial overlayers of 2,3,6,7,10,11-hexamethoxytriphenylene grown on graphene/SiC using Fourier transform photocurrent spectroscopy, photoluminescence, Raman spectroscopy, angle-resolved photoemission spectroscopy, and surface-sensitive microscopy. We resolve a vibronic manifold consistent with Davydov splitting arising from the *P*6_3_/*m* crystal symmetry, which lifts the highest occupied molecular orbital (HOMO)–lowest unoccupied molecular orbital (LUMO) degeneracy into bright and dark excitonic branches. Using a tight-binding model parametrized by experiment, we determine the intermolecular coupling, polarization energy, Huang–Rhys factor, and Herzberg–Teller corrections to the Franck–Condon model. The results indicate polaron-mediated relaxation into the lower-energy branch, consistent with Kasha’s rule. The developed graphene-supported molecular heterostructure provides a scalable platform for studying dark excitons and vibronic coupling in organic quantum materials.

## Graphical abstract

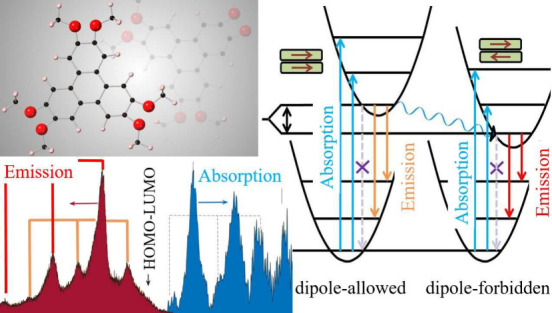

The simulation of open quantum systems, where electronic degrees of freedom are coupled to a complex bosonic environment, remains a frontier challenge in both condensed matter physics and quantum information science.^1–3^ Central to this challenge is the Holstein Hamiltonian,^4,5^ which describes the nonadiabatic dynamics of excitons and polarons—phenomena that are often computationally intractable in the strong-coupling regime.^6^ While synthetic quantum simulators such as trapped ions and superconducting circuits approximate Holstein-type models,^7–9^ experimentally accessible molecular platforms remain limited by structural disorder and fabrication-induced contamination.^10^ Here, we overcome this limitation using a streamlined nanofabrication and dioxolane cleaning protocol that restores epitaxial graphene on SiC to near-ultrahigh-vacuum (near-UHV) structural quality, as verified by low-energy electron diffraction (LEED) and low-energy electron microscopy (LEEM).

Parallel to achieving interfacial purity, the extreme photosensitivity of organic semiconductors^11,12^ necessitated a dynamic Raman acquisition protocol to prevent photodegradation.^13^ By employing submilliwatt excitation and rapid lateral scanning, we resolved previously undocumented vibrational modes essential for parametrizing the system’s vibronic coupling constants. Crucially, the resulting pristine interface allows the epitaxial graphene lattice to serve as a critical structural template that dictates the macroscopic coherence of the molecular overlayer.^14^ While growth on metallic, or dielectric substrates^15^ typically exhibits randomized azimuthal distribution, our LEED analysis confirms that 2,3,6,7,10,11-hexamethoxytriphenylene (HMTP) growth on graphene results in an exceptionally well-ordered phase^16,17^ locked to two discrete, symmetry-equivalent orientations. This template-induced alignment provides a route for resolving the fundamental vibronic structure^18–20^ and transition-dipole orientations of the molecular ensemble. Specifically, this crystalline order reveals the signature of Davydov splitting—a symmetry-breaking intermolecular exchange interaction^21^ that generates an upper bright and a lower dark excitonic branch. Leveraging the spectral resolution of this pristine interface, we synthesize results from Fourier transform photocurrent spectroscopy, angle-resolved photoemission spectroscopy (ARPES), and molecular dynamics (MD) to extract the fundamental constants of the Holstein Hamiltonian with unprecedented precision. We quantify the splitting strength (*J*), Huang–Rhys factors (*S*), and relative contributions of Franck–Condon (FC) versus Herzberg–Teller (HT) coupling mechanisms. Our findings demonstrate that the HMTP/graphene heterostructure operates in a regime where intermolecular electronic coupling is comparable to the vibrational relaxation energy, providing a rigorous testbed for nonperturbative theoretical models.^4,6^ By mapping the relevant phonon modes, we establish a benchmark platform for studying dissipation and electronic coherence in vibronically coupled molecular systems.

## HMTP Growth on Ultraclean Graphene

The HMTP/graphene heterostructures were prepared using a nanofabrication and surface-cleaning protocol designed to recover a graphene template with UHV-equivalent surface quality after device processing; see the Supporting Information (SI). The atomic-scale ordering of the active device regions was confirmed by LEED, LEEM, and scanning tunneling microscopy (STM), while complementary X-ray diffraction measurements verified the thermal stability of the graphene template and metallic contacts during the elevated-temperature outgassing procedure required for UHV characterization (see the SI).

In Figure 1a, LEED displays sharp diffraction spots and a low diffuse background in the complete area of the graphene electrode, confirming an atomically clean surface successfully recovered from resist residues postnanofabrication. The SiC(0001) diffraction spots are visible (orange arrows), alongside the 6-fold symmetric (1 × 1) spots characteristic of the epitaxial graphene lattice (yellow arrow) and the ( $6\sqrt{3}\times 6\sqrt{3}$ )­R30° surface reconstruction (violet arrows). This uniformity is corroborated by LEEM (Figure 1d), which reveals a high-contrast, homogeneous surface entirely devoid of the stochastic speckle contrast typical of cross-linked polymer remnants.^10^ The weak streaks observed in LEED (blue arrow in Figure 1a) are attributed to the microstripe morphology of graphene. Following submonolayer deposition, the LEED pattern (Figure 1b) reveals two additional sets of 6-fold symmetric diffraction spots. These sets originate from two discrete, symmetry-equivalent alignments of the HMTP lattice with respect to the underlying graphene. The molecular packing within the HMTP crystal contains two inequivalent molecular positions per unit cell,^32^ consistent with the *P*6_3_/*m* symmetry used in the vibronic analysis and tight-binding model (see the SI). The corresponding LEEM image (Figure 1e) shows the initial formation of HMTP aggregates (blue arrows), whereas such granulation is absent prior to HMTP deposition (red arrow in Figure 1d). We note that the bright stripes (green arrows) originate from charging of the SiC substrate, where graphene was removed during oxygen plasma etching. To confirm the atomic structure, STM and its fast Fourier transform (FFT) are presented in parts f and c of Figure 1, respectively. The FFT confirms a lattice constant of *a* = 1.2(1) nm, in good agreement with both the LEED diffraction pattern and the HMTP structural model employed in the data analysis (see the SI). The presence of only one hexagonal set in the FFT—compared to the two sets in LEED—is attributed to the localized probe area of STM, whereas LEED averages over larger domains containing both orientations. Building upon this structurally ordered interface, we deposited 50 nm HMTP films for optical and electronic characterization. The growth of the thicker HMTP films follows the orientational templating established in the first molecular layer, resulting in partially ordered epitaxial domains consistent with our previous study.^17^

**Figure 1:**
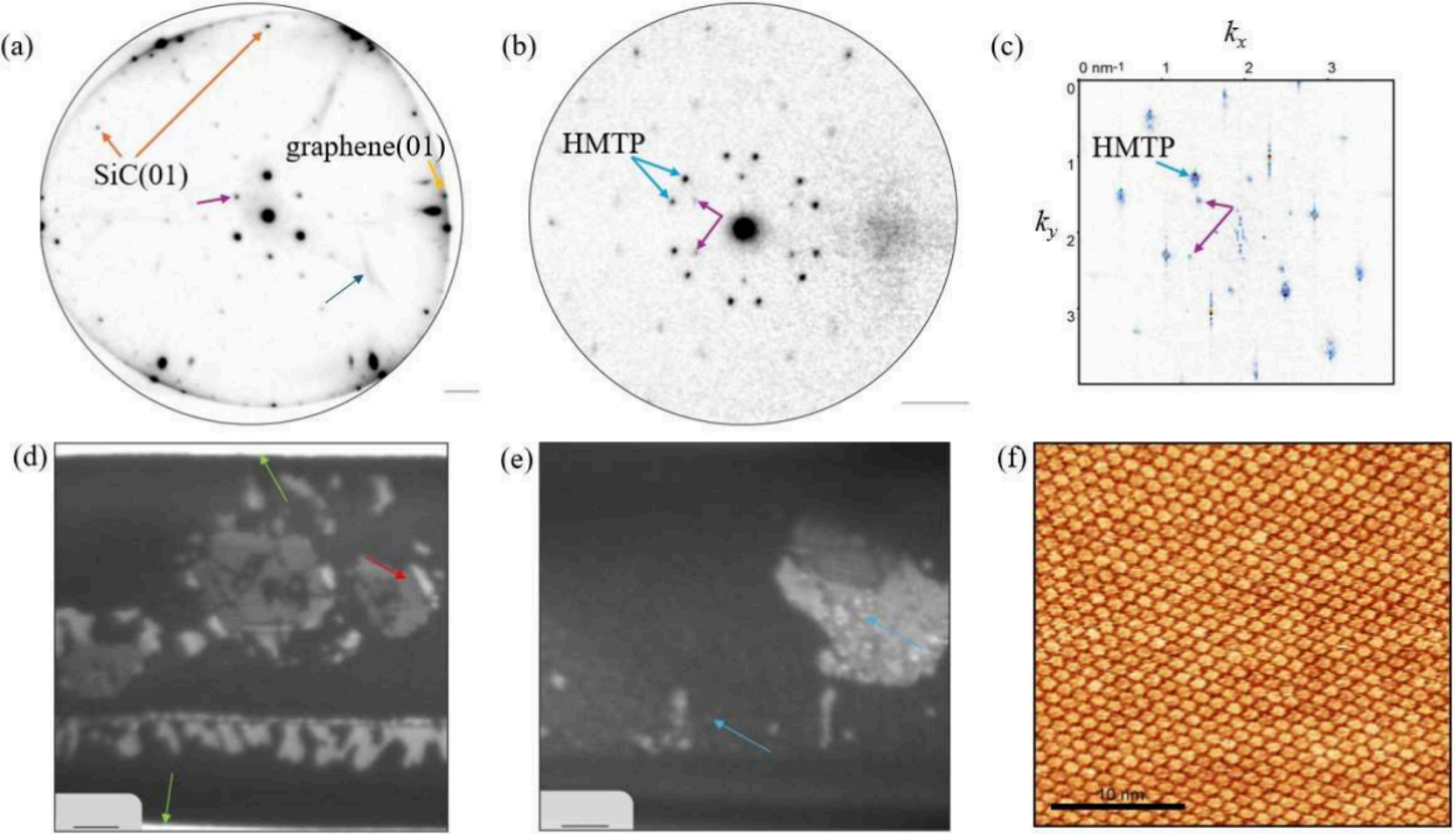
Structural characterization of HMTP submonolayers. LEED pattern of the graphene interdigitated device following the postnanofabrication cleaning procedure (a) before and (b) after HMTP submonolayer growth. The sharp diffraction spots in part (a) confirm the removal of polymer residues. (c) Numerical FFT of the STM image in part f, showing reciprocal space points consistent with the LEED symmetry in part b. (d) LEEM image of a 3-μm-wide graphene electrode before and (e) after HMTP deposition. Scale bars represent 0.5 μm. (f) High-resolution STM image of the HMTP layer on an unpatterned graphene substrate, revealing the molecular self-assembly.

As illustrated by the optical micrograph (Figure 2a), scanning electron microscopy (SEM; Figure 2b), and detailed atomic force microscopy (AFM) in the SI, the growth kinetics differ markedly among graphene, gold interdigital contacts, and the intercontact area. AFM height images (Figure 2c,d) further reveal distinct morphological regimes; on the graphene areas, HMTP maintains a higher degree of structural coherence, whereas growth on gold and SiC appears to be more disordered. This substrate-dependent morphology highlights the influence of the underlying substrate on the structural coherence and molecular ordering of the HMTP films.

**Figure 2:**
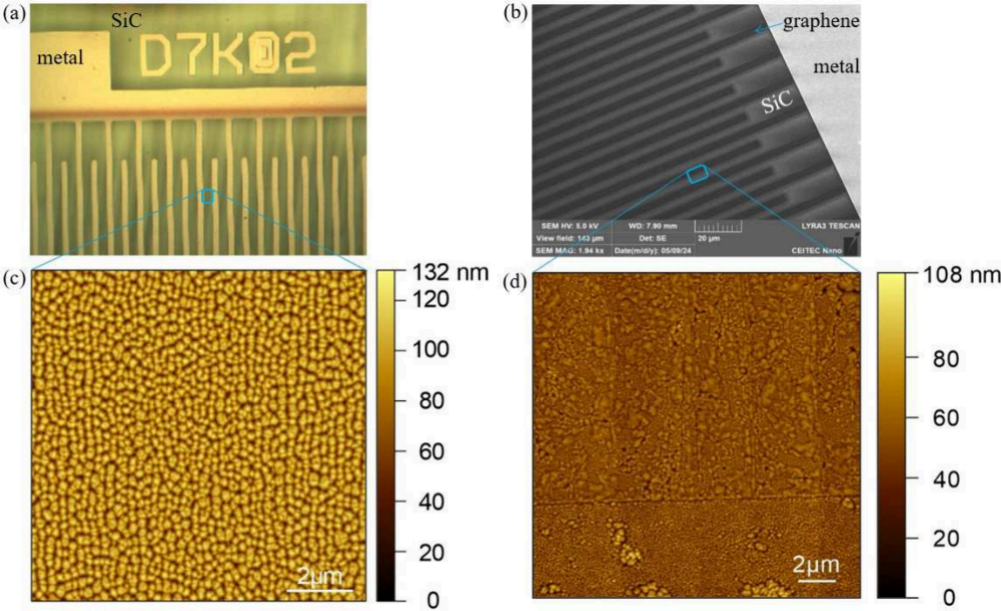
Morphological characterization of HMTP-based devices. (a) Optical micrograph of a Fourier transform photocurrent device featuring a prefabricated metal interdigitated electrode array. (b) SEM image of interdigitated contacts patterned from epitaxial graphene on SiC. (c) AFM topography of a 50-nm-thick HMTP film deposited onto the metal-electrode platform shown in part a. (d) AFM topography of the 50 nm HMTP film at the interface between the graphene contact (upper region) and the bare SiC substrate (lower region).

## Vibrational Modes and Molecular Dynamics

The vibrational landscape of the HMTP overlayer was characterized using a specialized Raman acquisition protocol designed to mitigate the extreme photosensitivity of the molecular species.^11,12^ To isolate the intrinsic molecular signatures from the dominant carbonaceous features of the substrate, measurements were performed on bare SiC regions (Figure 3a); this avoids spectral overlap with the graphene D and G peaks, which otherwise obscure the characteristic HMTP lattice modes.

**Figure 3:**
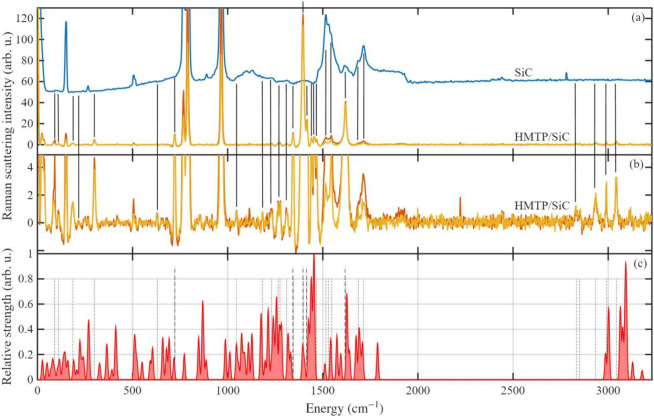
Vibrational characterization and MD validation. (a) Raman spectra of HMTP compared to the bare substrate (λ_exc_ = 532 nm). Spectra from the SiC substrate (blue) and the HMTP film at two distinct spatial locations (red and orange) are shown. (b) Magnified view of the low-intensity region, highlighting the subtle molecular vibrational features. Vertical solid lines denote characteristic HMTP modes; peaks overlapping with the blue SiC reference represent substrate phonon modes. (c) Calculated VDOS of HMTP obtained from MD simulations. Simulated transitions are convoluted with a Gaussian broadening function to match experimental resolution. Vertical dashed and dotted lines correlate the primary and secondary experimental modes with the simulated VDOS peaks.

To prevent photodegradation,^13^ Raman measurements were performed in the milliwatt excitation regime with a laser spot size of approximately 1 μm and excitation wavelength λ_exc._ = 532 nm. The full vibrational fingerprint was recovered by *x*–*y* Raman mapping and spatial averaging. By rapidly scanning a 30 × 30 μm^2^ area and spatially averaging the resulting 900 spectra, we achieved the high signal-to-noise ratio necessary to resolve low-intensity Raman modes. The reproducibility between distinct sample areas confirms the structural uniformity of the molecular aggregates. To support these experimental signatures, we calculated the vibrational density of states (VDOS) using MD simulations^33^ (Figure 3c). The simulation utilized a unit cell containing two inequivalent HMTP molecular positions, as identified in our structural analysis with periodic boundary conditions used to emmulate the long-range order of the molecular crystal. The calculated vibrational energies exhibit excellent agreement with experimental data—typically within 10–20% for MD.^34^ A comprehensive summary of the identified Raman modes, their relative intensities, and tentative assignments is provided in Table 1.

**Table 1:** Raman Peak Positions, Intensities, and Tentative Assignments for HMTP on SiC/Graphene

frequency (cm^–1^)	intensity (a.u.)	suggested assignment	ref
88	1.7	lattice/intermolecular phonon	^22^,^23^
110	1.1	lattice/intermolecular phonon	^22^,^23^
185	1.4	whole-molecule skeletal bending	^24^
297	4.4	methoxy (−OCH_3_) deformation	^25^,^26^
632	0.8	in-plane phenyl ring deformation	^27^
722	9.6	in-plane phenyl ring deformation, out-of-plane c–h deformation	^27^,^28^
1047	1.1	C–O–C stretching	^29^
1183	0.5	C–H in-plane bending	^29^
1232	1.1	ring–methoxy stretching	^29^
1266	1.4	ring–methoxy stretching	^29^
1274	1.4	ring–methoxy stretching	^29^
1310	1.1	softened Kekulé distortion in S_1_/C–O–C stretching	^30^
1343	11	Kekulé distortion (FC active, 168 meV)	^30^
1396	120	C–C stretching	^31^
1415	24	symmetric CH_3_ deformation	^29^
1438	5.7	CH_3_ deformation	^29^
1457	7.8	CH_3_ deformation	^29^
1470	5.7	asymmetric CH_3_ deformation	^29^
1514	4.1	aromatic C=C stretch	^29^
1529	6.0	aromatic C=C stretch	^29^
1544	5.7	aromatic C=C stretch	^29^
1617	42	primary aromatic C=C stretch	^29^
1685	1.7	combination band/overtone	
1714	3.5	combination band/overtone	
2832	0.8	2× symmetric CH_3_ Deformation	^29^
2851	0.8	symmetric CH_3_ stretching	^29^
2938	1.4	2× asymmetric CH_3_ deformation	^29^
2990	2.3	asymmetric CH_3_ stretching	^29^
3045	3.5	aromatic C–H stretching	^29^

The vibrational spectrum of HMTP is dominated by two strong features at 1396 and 1617 cm^–1^, corresponding to the aromatic ring stretching (C–C and C=C vibrations, respectively). The weaker mode at 1343 cm^–1^, assigned to the Kekulé distortion,^30^ acts as the primary mediator for electron–phonon coupling. Finally, the even weaker feature at 1310 cm^–1^ is attributed to the softening of this Kekulé mode in the S_1_ excited state, and it might also be contributed by the C–O–C stretching of the methoxy groups.^29^

## Electronic Structure and Excitonic Manifold

The excitonic landscape of the HMTP–graphene interface was probed using room-temperature photoluminescence (PL) and absorption spectroscopy (Figure 4). PL was excited at λ_exc_ = 355 nm using low excitation power densities to minimize the photoinduced degradation (laser spot size ≈10 μm; excitation power 10–20 μW). Absorption profiles were extracted via Fourier transform photocurrent spectroscopy using the underlying SiC substrate as a highly sensitive internal probe. By normalizing the photocurrent response of the functionalized device against the pristine SiC reference, we resolved the molecular absorption features. To account for minor setup variations during sample transfer, a smoothly varying spline background was subtracted; we verified that this procedure preserved the integrity of the spectral line shapes without introducing artificial optical responses. Both the PL and absorption spectra exhibit a striking mirror symmetry around the HMTP monomer highest occupied molecular orbital-to-lowest unoccupied molecular orbital (HOMO–LUMO) transition, characterized by two distinct sets of transitions: a primary, high-intensity manifold and a secondary, weaker set of satellite peaks (Table 2). To contextualize these optical signatures within the broader electronic structure, we performed ARPES on the HMTP overlayer (Figure 5). ARPES measurements were performed at room temperature using a He I discharge source. The dispersion is presented along the Γ–*K* direction of graphene reciprocal space. The photoemission features at −0.86 and −2.40 eV appear only after HMTP deposition and are therefore attributed to electronic states of the HMTP overlayer. Analysis of these two major valence levels was conducted using both multipeak Gaussian fitting and a model-unbiased center-of-mass (COM) integration to track the energy centroids (Figure 5b,c).

**Figure 4:**
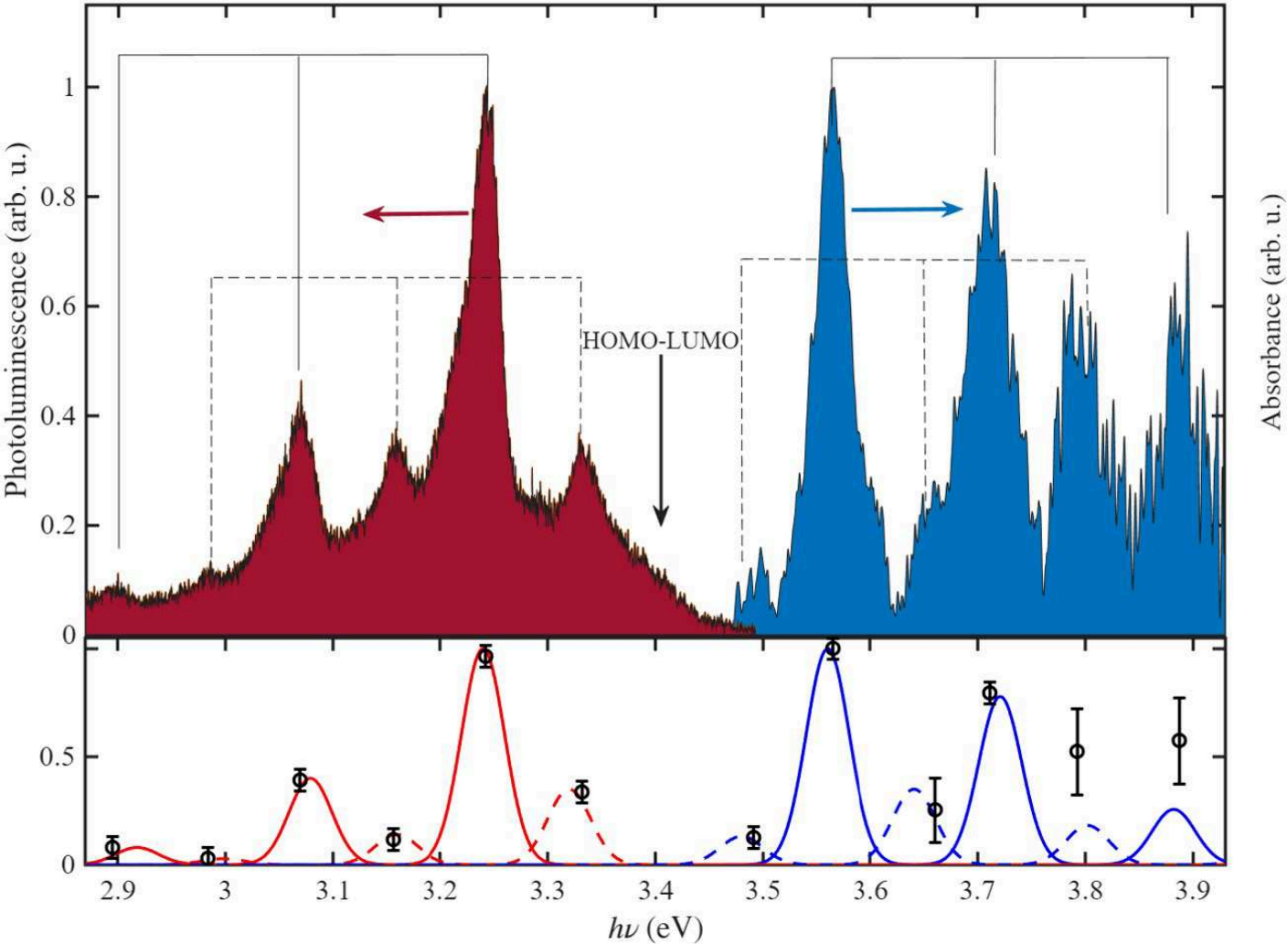
(Top) Experimental PL (red shaded area) and absorption (blue shaded area) spectra. Solid vertical lines indicate the vibrational manifold of the primary Davydov branch, while dashed vertical lines denote the minor (secondary) branch. (Bottom) Theoretical simulation based on a FC model incorporating HT corrections. Solid and dashed curves represent the calculated transitions for the main and minor manifolds, respectively. The model is benchmarked against experimentally extracted peak positions and intensities (black open circles with error bars). The unperturbed monomer HOMO–LUMO transition (*E*_monomer_ = 3.4 eV) is indicated for reference.

**Figure 5:**
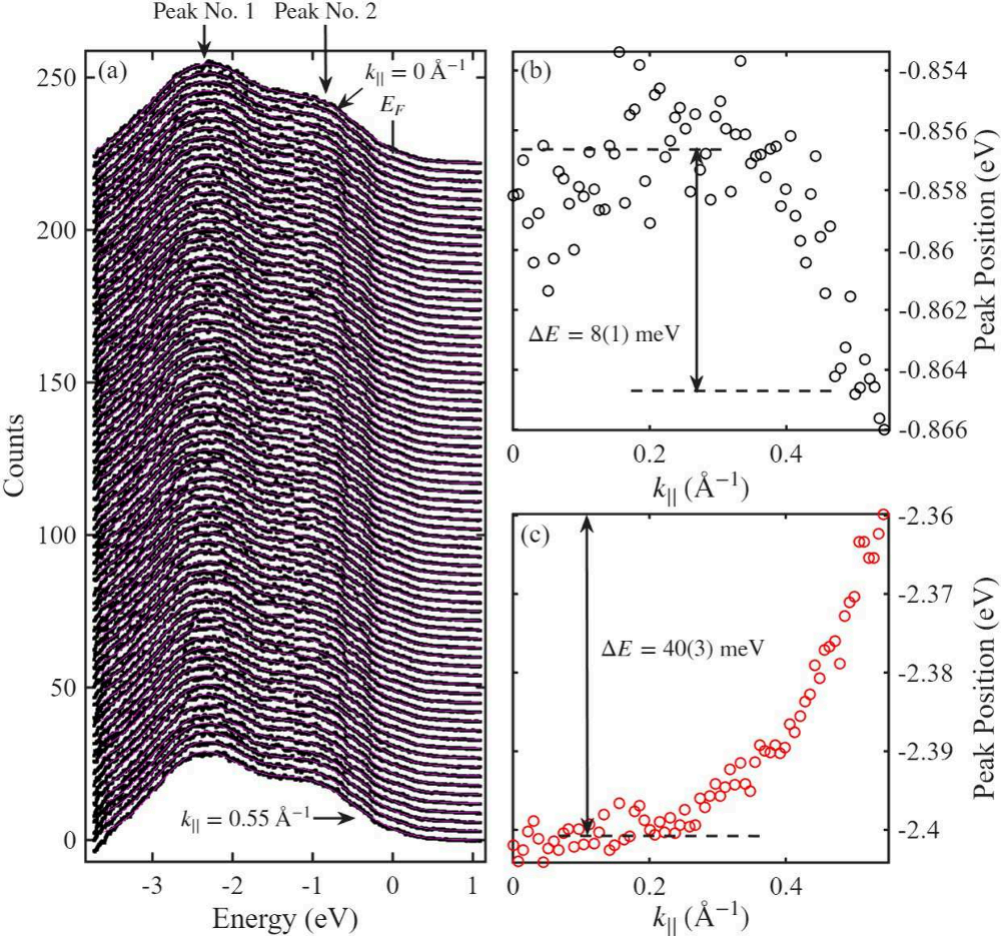
Electronic band structure and binding energy analysis of HMTP on graphene. (a) ARPES intensity map acquired along the Γ–*K* high-symmetry direction of the underlying graphene lattice; the energy axis is referenced to the Fermi level (*E* – *E*_F_ = 0 eV). (b and c) Detailed line-shape analysis of the two primary photoemission features located at (b) *E* – *E*_F_ = −0.86 eV and (c) *E* – *E*_F_ = −2.40 eV. Discrete peak positions were extracted using a COM integration method to track the dispersion (or lack thereof) across the sampled *k* space.

**Table 2:** Experimental PL and Absorption Peak Positions and Assignments (Monomer ZPL = 3.40 eV)

transition type	energy (eV)	intensity (a.u.)	assignment
Photoluminescence			
main set (1–0)	3.24	0.95	lower branch (S_1_)
main set (2–0)	3.07	0.39	first phonon replica
main set (3–0)	2.90	0.08	second phonon replica
minor set (1–0)	3.33	0.33	upper branch (S_2_)
minor set (2–0)	3.16	0.12	first phonon replica
minor set (3–0)	2.98	0.03	second phonon replica
Absorption			
main set (1–0)	3.57	0.99	upper branch (S_2_)
main set (2–0)	3.71	0.78	first phonon replica
main set (3–0)	3.89	0.57	second phonon replica
minor set (1–0)	3.49	0.12	lower branch (S_1_)
minor set (2–0)	3.66	0.25	first phonon replica
minor set (3–0)	3.79	0.52	second phonon replica

The resulting dispersion is remarkably flat, with a maximum bandwidth (BW) of only ≈40 meV. Within a tight-binding framework (see the SI), where BW = 13*t* for the HMTP crystal geometry, we estimate the intermolecular electronic coupling (*t*) to be on the order of 1–5 meV. This small electronic bandwidth—being significantly lower than the characteristic vibrational energies (10–200 meV) identified via Raman spectroscopy—places the HMTP/graphene system in the strong electron–phonon coupling regime. In this limit, the electronic excitations are expected to be strongly renormalized by lattice vibrations, motivating a description in terms of quasi-discrete vibronic states rather than delocalized Bloch waves.

## Interpretation of the Vibronic Structure

The symmetry of the absorption and emission profiles relative to the monomer transition suggests a dominant FC mechanism, modulated by the packing of the HMTP lattice. Given that the intermolecular electronic coupling (*t* ≈ 5 meV) is significantly smaller than the polarization energy (*P* ≈ 120 meV), we describe the system within a framework of quasi-discrete vibronic levels in the strong-coupling regime.^3^ Consistent with previous structural studies,^32^ the HMTP unit cell contains two inequivalent molecular sites. The LEED/STM measurements discussed above primarily characterize the epitaxial ordering of the first HMTP layer on graphene, whereas the optical response originates from multilayer HMTP films whose crystal packing follows the *P*6_3_/*m* structure shown in Figure 2 and is described in the SI. Taken together, the observed two interleaved vibronic progressions in PL and absorption, the strong electron–phonon coupling inferred from Raman spectroscopy and ARPES, and the presence of two inequivalent molecular sites motivate a tentative interpretation in terms of Davydov-split excitonic branches. Within this interpretation, the crystal packing would lift the degeneracy of the HOMO–LUMO transition into two Davydov-split branches separated by 2*J* = 90 meV (Figure 6),^21^ corresponding to bonding and antibonding excitonic states. The observed alternating sequence of strong and weak PL and absorption peaks is difficult to reconcile with a single FC vibronic progression, which would generally produce a smoother vibronic envelope even in the presence of HT corrections. Although HT coupling can modify the relative vibronic intensities and partially suppress selected replicas, it does not readily account for the experimentally observed alternating sequence of strong and weak peaks. Instead, the spectra are more naturally described by two vibronic progressions associated with the lower and upper Davydov branches. However, the polarization-resolved PL measurements presented in the SI exhibit only weak anisotropy. While this observation is consistent with the *P*6_3_/*m* crystal symmetry and domain averaging, it does not exclude alternative interaction mechanisms. We therefore consider the Davydov-splitting scenario to be the most plausible interpretation of the present data, while recognizing that alternative explanations cannot be fully excluded.

**Figure 6:**
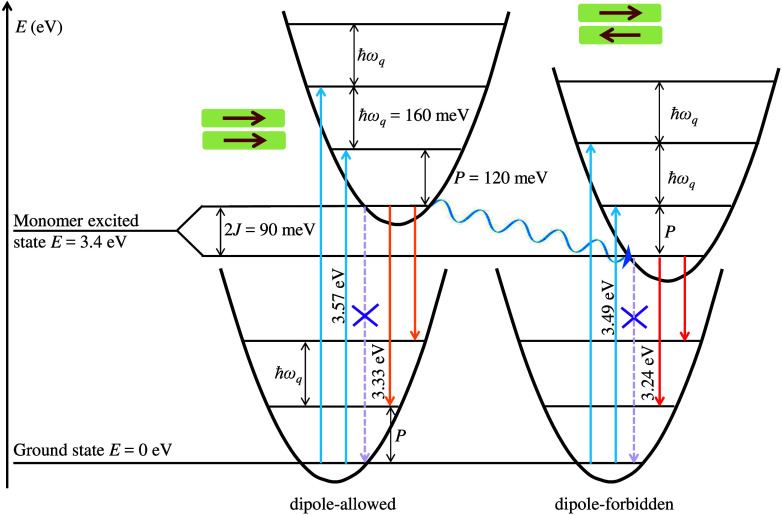
Configurational coordinate diagram of the HMTP electronic states. Potential energy parabolas for the ground state (S_0_) and the proposed Davydov-split excited states (S_1_). Vertical blue and orange/red arrows denote absorption and emission transitions, respectively. The relaxation pathway from the upper (bright) to the lower (dark/polaron) branch is indicated by the wavy line. The forbidden transitions 0–0 are depicted by the violet dashed lines (crossed).

## Absorption, Emission, and the HT Correction

Following the proposed interpretation, the primary absorption peak centered at 3.57 eV is assigned to the dipole-allowed transition to the upper Davydov branch (1–0 transition). Higher-energy peaks at 3.71 and 3.89 eV represent subsequent phonon replicas (2–0 and 3–0), characterized by the emission of ≈160(20) meV phonons—identified in our Raman data as the Kekulé distortion of HMTP aromatic rings. A secondary, lower-intensity set of absorption peaks (dashed lines, Figure 4) is attributed to transitions to the lower Davydov branch. While these transitions are nominally symmetry-forbidden, the strong polaron coupling distorts the lattice sufficiently to transfer oscillator strength into these dark states. We find that the intensities of the main manifold follow a standard FC progression with a relatively small HT correction α = 0.2(1); see the SI for derivation. However, the minor manifold requires a larger HT correction α = 0.8(3) to describe the anomalous intensity distribution

1 $$I_{n}\left(S,\alpha \right)\propto \frac{S^{n}}{n!}{\left(1+\alpha \frac{n\pm S}{\sqrt{S}}\right)}^{2}$$

where *S* ≈ 0.4 is the Huang–Rhys factor and α accounts for the coordinate dependence of the transition dipole moment and + (−) stands for emission (absorption). This HT contribution highlights the non-FC effects introduced by the intense exciton–phonon coupling in the ordered overlayer.^19^

In contrast to the absorption spectra, the emission spectra are well described, within the present experimental resolution, by a FC progression with negligible HT correction (α ≈ 0). Following lattice relaxation to the *n* = 0 vibronic state, polarons accumulate in the lower Davydov branch (blue wavy line, Figure 6). The strongest PL transition at 3.24 eV is assigned to the 1–0 emission from this lower-energy branch, consistent with Kasha’s rule,^35^ followed by the corresponding 160(20) meV phonon replicas. We also resolve a minor set of peaks starting at 3.33 eV, which we attribute to hot PL from the upper Davydov branch.

## Quantitative Spectral Modeling

For strongly vibronically coupled molecular aggregates, absorption and emission line shapes are often treated in the literature using effective vibronic Hamiltonians, including Frenkel–Holstein-type approaches.^36–38^ These descriptions highlight that the observed spectral intensities are governed not only by electronic coupling but also by FC factors, exciton–phonon coupling, and non-Condon corrections. In the present work, we adopt a reduced experimentally constrained FC/HT model rather than a full first-principles treatment of the complete HMTP crystal. The transition energies are fixed by the Raman-active vibrational modes and the experimentally observed PL/absorption peak positions, while the relative intensities are obtained from displaced-oscillator wave functions including a first-order coordinate dependence of the transition dipole moment. The derivation (in the SI) provides an effective vibronic framework for the absorption and emission manifolds and shows explicitly how the Huang–Rhys factor and HT correction enter the spectral intensities.

To illustrate the proposed vibronic framework, we modeled the PL and absorption spectra using a series of Gaussian functions with a fixed broadening of σ = 20 meV to account for the experimental line widths. Within the proposed two-manifold interpretation, the total spectral response *A*(*E*) is expressed as a sum over two excitonic branches (*m* = ±) and their associated phonon manifolds:

2 $$A\left(E\right)=\underset{m=\pm }{\sum }\overset{\infty }{\underset{n=0}{\sum }}C_{m}I_{n}\left(S,\alpha \right)\;\exp\left(-\frac{{\left(E-E_{n,m}\right)}^{2}}{2\sigma^{2}}\right)$$

where the transition energies *E*_*n*,*m*_ are governed by the interplay of the monomer HOMO–LUMO gap, the polarization energy *P*, and the intermolecular exchange coupling *J*. Specifically, for absorption

3 $$E_{n,m}=E_{\mathrm{monomer}}+P\pm J+n\hbar \omega$$

and for emission

4 $$E_{n,m}=E_{\mathrm{monomer}}-P\pm J-n\hbar \omega$$

In this model, *P* represents the energy required to reconfigure the lattice upon exciton formation (the polaron shift), while *J* signifies the splitting between the bonding and antibonding branches. Table 3 summarizes the model parametrization. As shown by the red (PL) and blue (absorption) curves in the bottom part of Figure 4, this analytical approach provides a consistent description of the experimental data. The solid lines denote the primary dipole-allowed transitions, while the dashed lines represent the minor transitions whose intensities in absorption are dictated by the HT-corrected Huang–Rhys factors.

**Table 3:** Parameters for the Vibronic Model of HMTP on SiC/Graphene

category	parameter	value	units/notes
electronic origin	monomer energy (*E*_monomer_)	3.4	eV
crystal shifts	polarization shift (*P*)	±120	meV
	excitonic coupling (*J*)	±45	meV
vibronic scaling	phonon energy (*ℏω*)	160(20)	meV
	phonon quanta (*n*)	0, 1, 2	integer
	Huang–Rhys factor (*S*)	0.4(2)	dimensionless
	line broadening (σ)	20	meV (Gaussian)
branch indices	branch index (*m*)	±1	+1, bright; −1, dark
	weighting factor (*C*_*m*_)	Var.	relative branch population
HT correction (α)	main PL branch (α_PL,main_)	0	vibronic-induced transition
	minor PL branch (α_PL,minor_)	0	allowed (hot) transition
	main Abs branch (α_Abs,main_)	0.2(1)	weak vibronic mixing
	minor Abs branch (α_Abs,minor_)	0.8(3)	forbidden (vibronic-induced)

We note that a single FC/HT progression based on eq 1 cannot account for the data. The Raman-constrained phonon energy reproduces the observed vibronic spacing within each manifold but leaves the second peak manifold unexplained, while forcing an apparent 80–90 meV progression is incompatible with the measured Raman spectrum and fails to reproduce the correlated PL/absorption intensities. In contrast, the two-manifold FC/HT model provides a consistent description of both the peak positions and relative intensities using parameters constrained by independent Raman, ARPES, and structural measurements. This experimentally constrained description is consistent with the broader Holstein framework of vibronically coupled molecular aggregates.

We demonstrated an atomically well-defined hybrid platform that bridges scalable nanofabrication with surface science characterization tools. Using a specialized nanofabrication and cleaning protocol, we recovered a UHV-equivalent graphene template on a functionalized device architecture and parametrized the Holstein Hamiltonian in a HMTP/graphene system through the combined analysis of LEED, STM, ARPES, and high-fidelity Raman spectroscopy. The proposed model of a Davydov-split excitonic manifold, modulated by strong exciton–phonon coupling, provides a plausible interpretation of the observed hierarchy of optical transitions. Within this framework, the dominant PL originates from the lower-energy branch following relaxation from the upper branch, whereas the weak higher-energy manifold is attributed to hot PL. These excitonic states may provide an attractive platform for studying excitonic dynamics and their potential relevance to solid-state quantum storage.

## Supplementary Material



## Data Availability

The data that support the findings of this paper are openly available.

## Notes

HMTP crystallographic unit cell; detailed nanofabrication and graphene cleaning procedures; thermal stability of device metallization; substrate-dependent HMTP morphology; Raman spectroscopy of HMTP on SiC, graphene, and powder samples; polarization-resolved PL; tight-binding derivation of the HMTP band structure; derivation of FC and HT vibronic intensities (PDF)
