# Supplementary material for: Vibronic and Excitonic Structure of a Template-Engineered Molecular-Graphene Heterostructure
Source: Nano Lett. 2026 Jul 21;26(30):9751–8. doi: 10.1021/acs.nanolett.6c01284 (PMC13450483; doi:10.1021/acs.nanolett.6c01284)
Supplement: Supplementary file 1 [file nl6c01284_si_001.pdf]

Supplementary Information

for

Vibronic and Excitonic Structure of a Template-Engineered Molecular-Graphene Heterostructure

J. Kunc[1, *], B. Morzhuk[1], V. Stará[2], D. Varshney[3], M. Shestopalov[1], K. Matějka[1], M. Rejhon[1], J. Novák[3], and J. Čechal[2]

[1] Charles University, Faculty of Mathematics and Physics, Institute of Physics, Ke Karlovu 5, Prague 2, CZ-121 16, Czech Republic

[2] CEITEC - Central European Institute of Technology, Brno University of Technology, Purkyňova 123, Brno, CZ-612 00, Czech Republic

[3] Masaryk University, Department of Condensed Matter Physics, Kotlářská 267/2, Brno, CZ-611 37, Czech Republic

*Email: jan.kunc@matfyz.cuni.cz

Table of Contents

1 Unit cell . . . S3

2 Detailed Nanofabrication Parameters . . . S3

2.1 Resist Stack Engineering . . . S3

2.2 E-beam Lithography and Optimization . . . S4

3 Structural Stability of Metallization upon Thermal Annealing . . . S5

4 Graphene and HMTP Growth and Substrate-Dependent Morphology . . . S6

4.1 Graphene Growth . . . S6

4.2 Submonolayer HMTP Growth . . . S7

4.3 Thin HMTP layers . . . S8

5 Macroscopic Device Alignment and HMTP Film Distribution . . . S8

6 Raman spectroscopy . . . S8

7 Polarization-Resolved Photoluminescence . . . S10

8 Tight-Binding Derivation of the HMTP Band Structure . . . S11

8.1 Hopping Vectors and Coordination . . . S11

8.2 Tight-Binding Hamiltonian . . . S12

8.3 Dispersion and Bandwidth . . . S13

9 Vibronic Transitions and Herzberg-Teller Corrections . . . S13

9.1 Displaced Harmonic Oscillator . . . S14

9.2 Franck-Condon Intensities S14
9.3 Herzberg-Teller Correction S14

Supplementary References S17

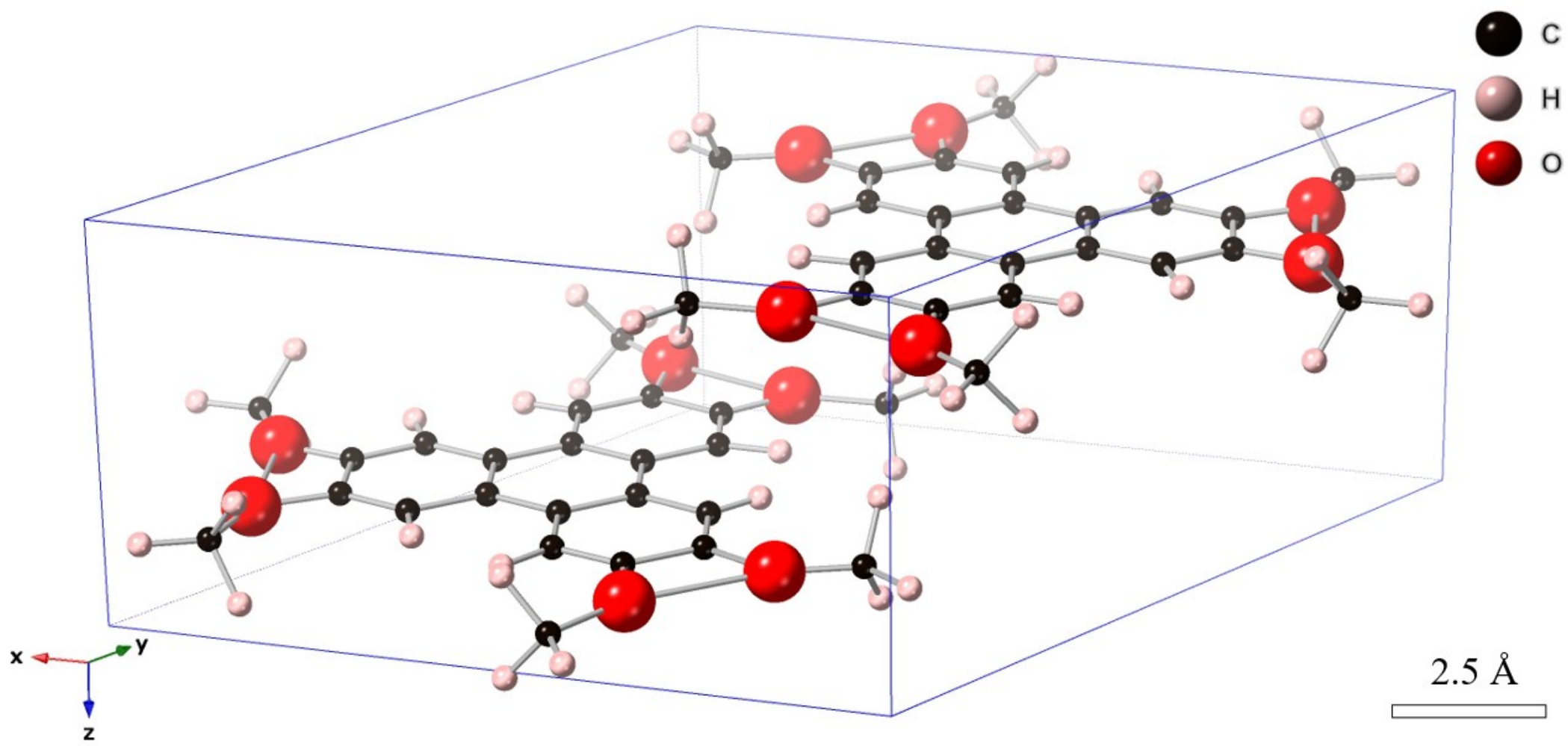

Figure S1: Crystallographic unit cell of 2,3,6,7,10,11-hexamethoxytriphenylene (HMTP) is depicted by a blue rhombic prism. The spheres represent carbon (black), oxygen (red), and hydrogen (white) atoms. The arrangement corresponds to the $P6_3/m$ space group symmetry.

Table S1: Step-by-step spin-coating parameters for the four-layer resist stack.

**Resist Layer**	**Spin Speed**	**Duration**	**Baking Temp. / Time**
PMMA (AR-P 679.04)	4000 rpm	65 s	200°C / 10 min
MMA (AR-P 617.08)	4000 rpm	65 s	200°C / 10 min
MMA (AR-P 617.08)	4000 rpm	65 s	180°C / 10 min
Electra AR-PC 5090.02	4000 rpm	65 s	85°C / 3 min

1 Unit cell

The unit cell of 2,3,6,7,10,11-hexamethoxytriphenylene (HMTP) used in the calculations of the vibrational density of states, Fig. S1.

2 Detailed Nanofabrication Parameters

2.1 Resist Stack Engineering

The four-layer resist stack was engineered to create a controlled sensitivity gradient that optimizes the undercut profile for edge-contact formation while protecting the graphene channel (Table S1). The MMA (AR-P 617.08) layers were baked at different temperatures to modulate their sensitivity to the electron beam. The MMA layer baked at 200°C becomes more sensitive than the upper MMA layer baked at 180°C. This creates a controlled lateral development rate, resulting in a stable, high-fidelity undercut necessary for clean metal lift-off. A bottom layer of PMMA (AR-P 679.04) is critical for graphene protection. Unlike MMA, this layer acts as a sacrificial barrier that

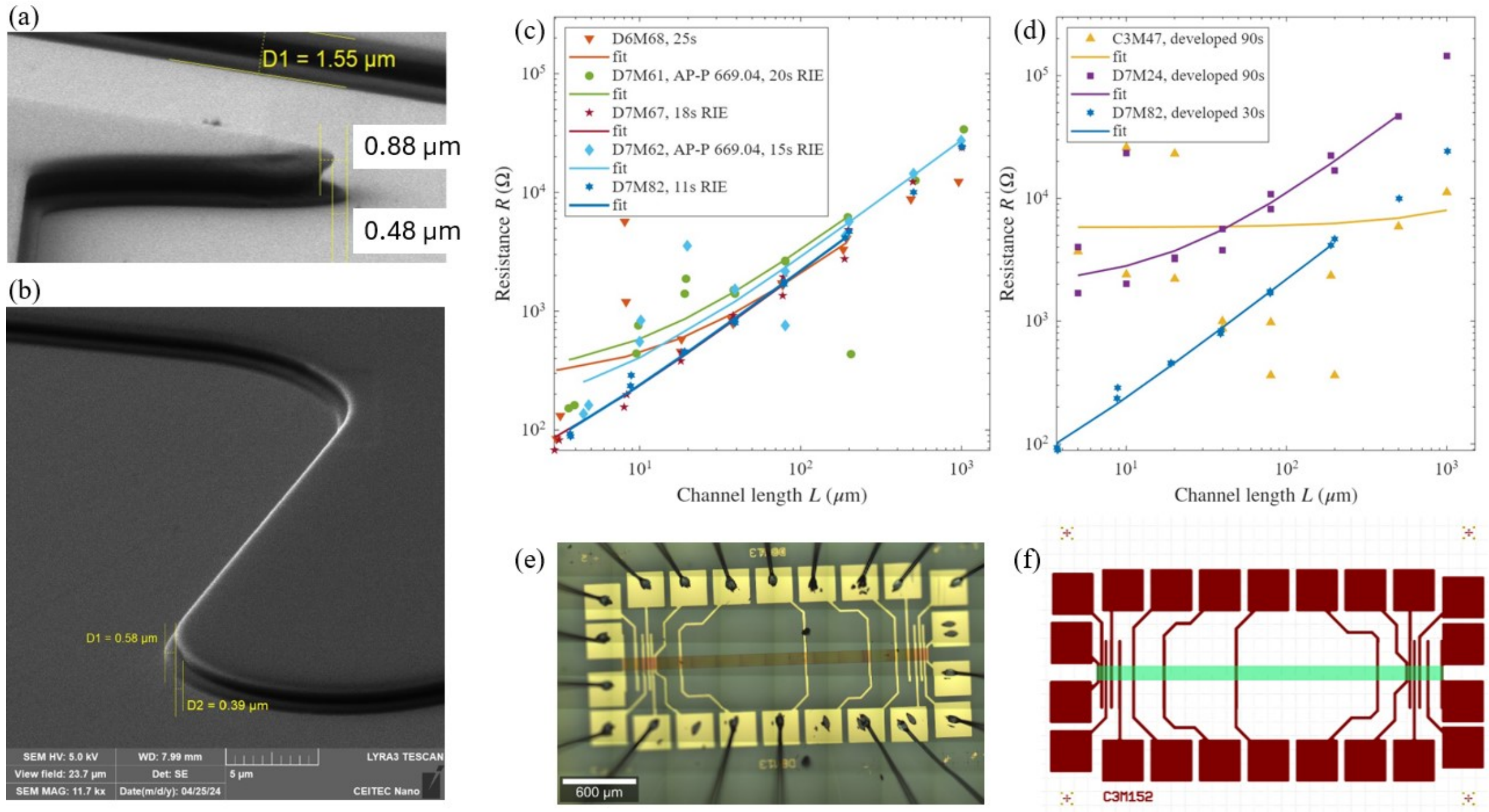

Figure S2: Nanofabrication workflow and process optimization for graphene edge-contact devices. (a) High-resolution SEM side-view ($85°$ tilt) of the developed four-layer resist stack (from the SiC/graphene to the top layer - PMMA/MMA/MMA/Electra), demonstrating the undercut profile required for metal lift-off. (b) Side-view SEM view of the developed resist over a large area. (c, d) Resistance vs. graphene channel length (TLM) for process optimization: (c) variation with oxygen plasma etching duration (11–25 s) and (d) comparison of resist development times (30 s vs. 90 s). (e) Optical micrograph of the wire-bonded Transfer Length Method (TLM) device. (f) GDS layout illustrating the two-step process for the TLM device fabrication: (red) first-step metallization and (green) graphene shaping via oxygen plasma etching.

protects the graphene from over-etching during the oxygen plasma step. Because oxygen plasma etching is not perfectly directional, the absence of this layer would allow the plasma to spread beneath the MMA undercut, over-etching the graphene beyond the intended contact area defined by the more directional metal evaporation.

2.2 E-beam Lithography and Optimization

Patterning was performed using a Raith 150-Two / Tescan Mira system at 20 kV with a beam current of 2–3 nA and a dose of 300 μC/cm^2 (step/line spacing: 50 nm). The electrical characteristics of the contacts were found to depend sensitively on both development and etching times. As shown in Fig. S2 (c), oxygen plasma etching longer than 25 s results in non-ohmic contacts due to excessive lateral etching. Our optimal window was found to be 11–15 s. Apart from the sequential development[S1], our single-step development in AR 600-56 was limited to 30 s to preserve the integrity of the PMMA support layer. Extending development to 90 s, Fig. S2 (d), causes the PMMA base layer to thin excessively and recede too far beneath the MMA undercut, disrupting the precise alignment of the metal evaporation with the graphene edge.

Table S2: Development and Cleaning Protocol for the All-Methacrylate Stack.

Process Step	Chemical/Medium	Duration / Conditions
Development:		
Electra Removal	DI Water	30 s
Resist Development	AR 600-56 (Methyl isobutyl ketone)	30 s
Stopper	Isopropyl Alcohol (IPA)	15 s
Rinse	DI Water	15 s
Drying	Compressed N_2	Until dry
Post-Fab Cleaning:		
Primary Soak	Acetone	2.5 h (Never-dry)
Rinse 1	IPA	30 min (Never-dry)
Rinse 2	DI Water	5 min (Never-dry)
Residue Removal	AR 600-71 (1,3-Dioxolane based)	1.5 h (Never-dry)
Final Rinse	DI Water	10 min (Never-dry)
Final Drying	Compressed N_2	High-purity flow

3 Structural Stability of Metallization upon Thermal Annealing

To recover the pristine graphene surface for LEED and LEEM characterization, the fabricated devices must undergo a UHV outgassing procedure at 500°C for 5 minutes. While the epitaxial graphene remains inherently stable at these temperatures, it is necessary to verify the structural integrity of the wirebonding pads and interdigitated metal contacts (Ti/Cu/Au stack) for the reference sample, to ensure that the device geometry remains intact for subsequent molecular deposition and electrical probing. As shown in Figure S3, X-ray diffraction (XRD) was employed to monitor the evolution of the metal stack—comprising 5 nm Ti, 220 nm Cu, and 20 nm Au—throughout the processing stages. X-ray coplanar diffraction ($\theta - 2\theta$) measurements were performed using a Rigaku SmartLab 3 X-ray diffractometer equipped with a rotating Cu anode (wavelength $\lambda = 0.154$ nm) and a five-circle goniometer. For this measurement, a monochromatized and collimated incident X-ray beam with a size of 0.2 mm was achieved using a parabolic multilayer mirror, followed by a 0.3 mm pinhole and a 0.2 mm collimator. The diffracted intensity was recorded using a two-dimensional detector (HyPix-3000) positioned 150 mm from the sample.

In the as-deposited state, distinct Bragg reflections corresponding to the (111) Au and (200) Cu planes are observed. Following the 500°C annealing step, the (111) Au peak exhibits a marked reduction in intensity and significant broadening, while the (200) Cu diffraction intensity increases. These spectral shifts are characteristic of inter-diffusional alloying at the Cu-Au interface, leading to the formation of a solid solution or disordered alloy phase.

Despite this interfacial alloying, the overall morphological integrity of the leads is preserved. This is evidenced by the persistent macroscopic definition of the contacts in optical micrographs and the successful nucleation of the molecular overlayer. Upon the deposition of HMTP on the annealed contacts, a new diffraction feature emerges at the position corresponding to the (002) reflection of the HMTP molecular crystal. This confirms that the annealed metal leads remain a suitable substrate for molecular assembly. We therefore conclude that while thermal processing

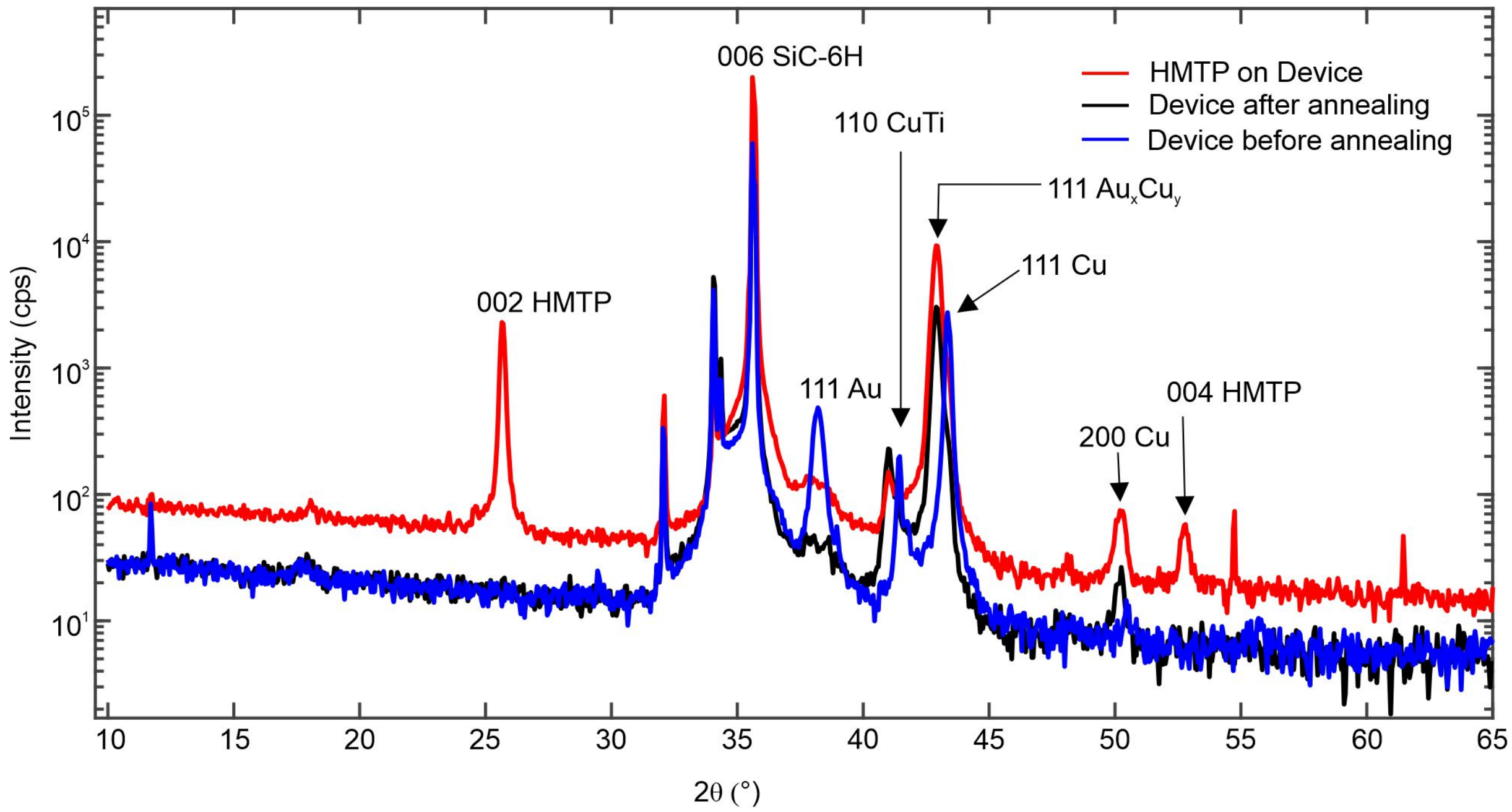

Figure S3: Structural evolution of the Fourier Transform photocurrent device during processing. XRD diffractograms of the metallic interdigitated electrode device at different fabrication stages: initial device (black), following UHV outgassing at 500°C for 5 min to remove adsorbed moisture (blue), and after 50 nm HMTP film deposition (red). Comparison of the black and blue curves reveals a broadening of the Au reflections and a slight shift/intensification of the Cu peaks, suggesting the onset of Au-Cu interfacial alloying during the thermal treatment. Despite these interfacial changes, the overall electrode geometry remains intact. The additional diffraction features in the red curve correspond to the crystalline phases of the HMTP layer.

induces a controlled Cu-Au alloying, it does not compromise the lithographic definition or the structural stability of the device contacts.

4 Graphene and HMTP Growth and Substrate-Dependent Morphology

4.1 Graphene Growth

Epitaxial graphene was synthesized by thermal decomposition of the Si-face of 4H- or 6H-SiC(0001) substrates[S2] (sourced from Coherent/II-VI Inc. and Wolfspeed/Cree Inc.). The substrates 5×5 mm^2 were cleaned in acetone, isopropanol, and deionized (DI) water prior to loading them into the growth furnace. The growth process followed a high-temperature annealing in a purified argon atmosphere (at a pressure of ≈ 1000 mbar) to regulate the silicon sublimation rate[S3,S4]. The thermal cycle consisted of sample outgassing and surface preparation at 800°C and 1100°C for 10 minutes each to ensure surface cleanliness and reconstruction. The final growth stage was taken at 1625-1650°C for 5 minutes. The precise growth temperature is calibrated according to the aging of the graphite crucible, as established in our previous optimization studies[S4,S5]. This calibration ensures

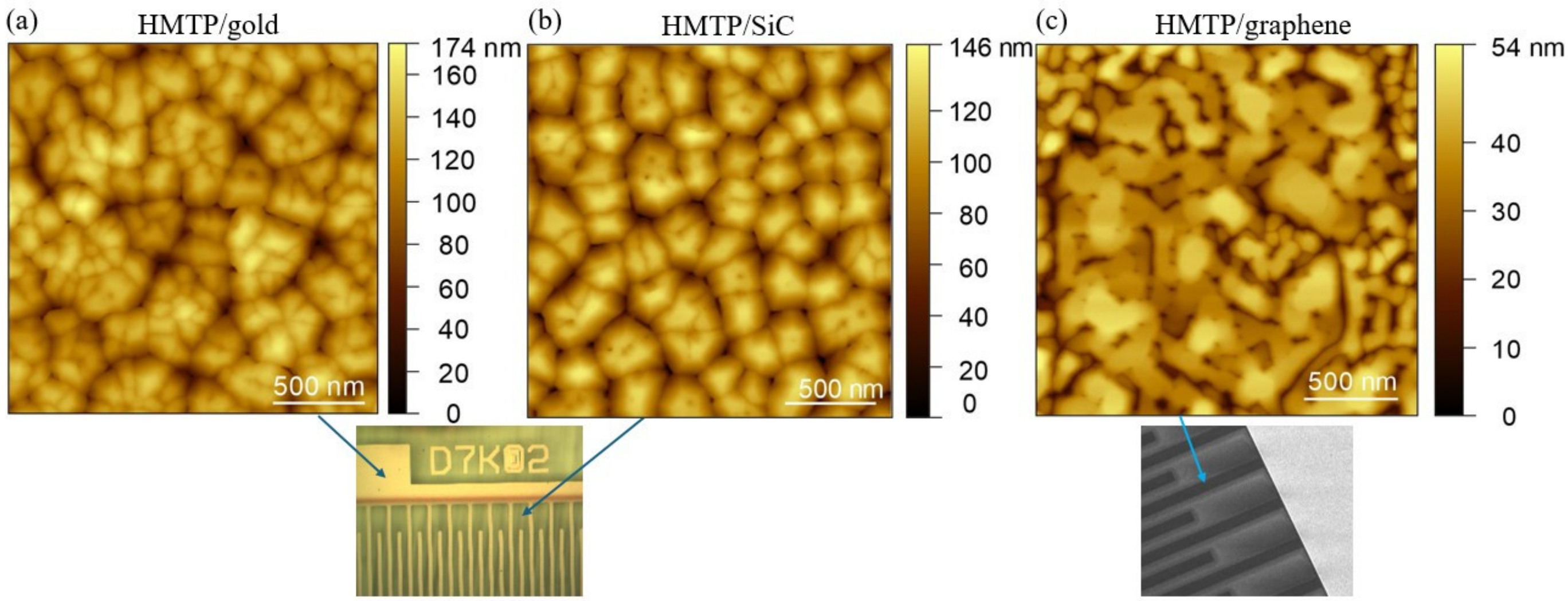

Figure S4: Morphology of HMTP films on different device regions. AFM topography of a 50 nm thick HMTP film deposited on the Fourier Transform photocurrent device. (a) Surface morphology on the gold-covered electrode area (20 nm Au / 220 nm Cu / 5 nm Ti metallization). (b) Surface morphology in the channel region between the electrodes (SiC substrate area). Differences in grain size and nucleation density between (a) and (b) reflect the distinct surface energies of the polycrystalline gold contact versus the underlying substrate.

the formation of a uniform monolayer with minimal bilayer coverage, providing the necessary atomically flat template for subsequent HMTP self-assembly.

4.2 Submonolayer HMTP Growth

All graphene samples (with and without contacts) were annealed at 530°C for 20 min in a preparation chamber with a base pressure of $2{\times}10^{-10}$ mbar. For thin film experiments, HMTP molecules were deposited in the LEEM chamber using an organic material evaporator (MBE Komponenten, Quad Cell OEZ40). The molecular powder (Merck) was kept in a resistively heated quartz crucible and operated at 170-190°C. The LEEM chamber design results in a deposition rate that is an order of magnitude lower than in thick-layer deposition. Room-temperature Scanning Tunneling Microscopy (STM) images were obtained on a commercial system, Aarhus SPECS, using the KolibriSensor and a base pressure in the chamber of $1{\times}10^{-10}$ mbar. Measurements were performed on a graphene/SiC sample without contacts using a constant current mode, the sample bias of 0.7 V, and the tunneling current of 50 pA. The contrast of the images was adjusted in Gwyddion software. Specs FE-LEEM P90 instrument was used to perform Low-Energy Electron Microscopy/Diffraction (LEEM/LEED) experiments (base pressure of $2{\times}10^{-10}$ mbar). The bright-field images were measured by collecting electrons from the central (0,0) beam. The diffraction patterns were taken from a surface area of $15 \times 10\ \mu\mathrm{m}^2$ or using a set of apertures that reduced the beam spot on the surface to 185 nm in diameter.

4.3 Thin HMTP layers

HMTP molecules were purchased from Merck. HMTP thin films with a thickness of 50 nm were deposited using an organic molecular beam evaporator (MBE Komponenten OEZ) equipped with a resistively heated quartz crucible operated at 175°C. The deposition was carried out in a deposition chamber with a base pressure of 5×10^{-10} mbar. Prior to deposition, the substrates were cleaned by annealing at 480°C for 5-15 minutes under ultra-high vacuum (UHV) to remove surface contaminants. The deposition rate, calibrated on pure single-layer graphene (SLG) samples using X-ray diffraction (XRD), was 5 Å/s. All the samples were kept at 25°C (room temperature) during deposition, and identical growth conditions were applied for all thin film preparations.

To investigate the influence of the underlying substrate on the growth of 50 nm thin HMTP layer, we performed high-resolution AFM topography on 2×2 μm regions of the functional device (Fig. S4). This scale allows for a quantitative comparison of the grain boundary density and nucleation kinetics across the different material interfaces. AFM measurements were performed using a Bruker Dimension Icon microscope operated in tapping mode, and the acquired data were analyzed using Gwyddion software[S6]. On the polycrystalline gold electrodes [Fig. S4(a)], the HMTP film forms a dense network of small, isotropic grains. Such a disordered landscape is expected to accelerate excitonic dephasing and localize transitions. In contrast, the morphology in the inter-electrode regions [Fig. S4(b)]—where the SiC is exposed—exhibits a distinct grain evolution. While more ordered than the growth on gold, it lacks the long-range azimuthal orientation observed on the pristine graphene template S4(c). This comparison confirms that the 1×1 graphene lattice is uniquely capable of suppressing stochastic nucleation and enforcing the structural coherence.

5 Macroscopic Device Alignment and HMTP Film Distribution

To confirm the spatial uniformity and precise alignment of the molecular overlayer with the underlying contact architecture, we performed large-area optical microscopy. The 50 nm thick HMTP film was deposited by thermal evaporation through a shadow mask, resulting in a well-defined molecular band across the active region of the device. As shown in Figure S5(a), the low-magnification micrograph reveals a clear contrast difference between the pristine substrate and the deposited HMTP band (shown by blue arrows). This macro-scale verification ensures that the interdigitated electrode array is fully integrated within the functional molecular region. At higher magnification [Fig. S5(b)], the distribution of HMTP molecular aggregates is visible across the active channel. The consistent optical contrast between the metal electrodes and the SiC regions confirms that the deposition protocol allows for continuous macroscopic coverage without compromising the lithographic definition of the device leads.

6 Raman spectroscopy

To verify that the Raman mode assignment used in the main text is not an artifact of the SiC-supported film, we compared Raman spectra of HMTP deposited on SiC, HMTP deposited on graphene, and HMTP powder consisting of molecular crystallites. The molecular arrangement is

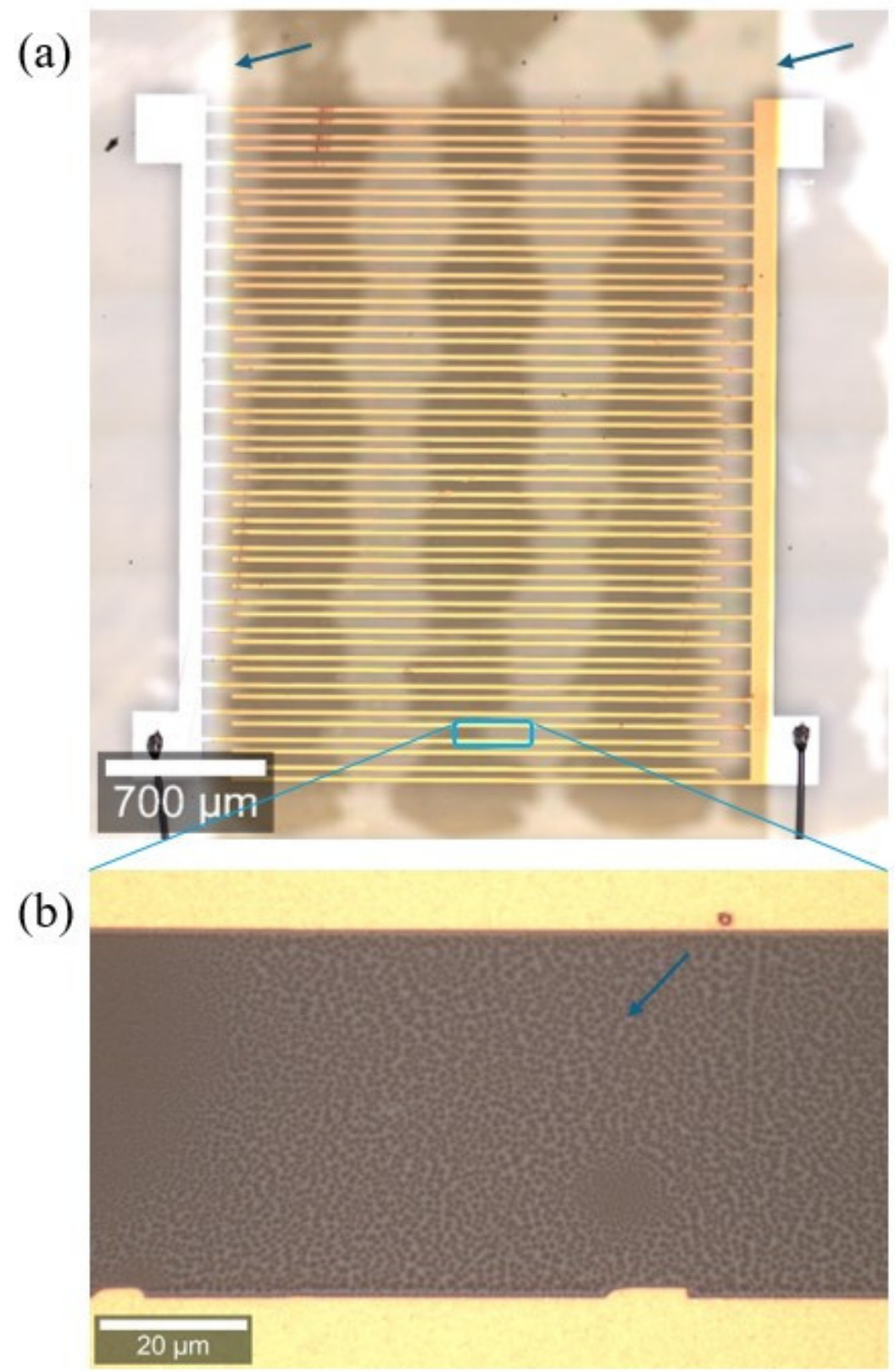

Figure S5: Large-scale device morphology and HMTP film distribution. (a) Low-magnification optical micrograph of the complete Fourier Transform photocurrent device. The dark transverse region (edges indicated by blue arrows) defines the 50 nm thick HMTP layer deposited via thermal evaporation through a shadow mask. (b) High-magnification optical micrograph of the active channel area. The visible contrast (representative features highlighted by the blue arrow in (b) confirms the distribution of HMTP molecular aggregates between the interdigitated metal electrodes, demonstrating uniform coverage across the device geometry.

not identical on the two substrates: HMTP on graphene is azimuthally ordered by the graphene lattice, whereas HMTP on SiC forms azimuthally disordered molecular domains. Despite this difference, the dominant Raman features are observed consistently across the different HMTP preparations within the experimental limitations. In the HMTP/graphene spectrum, several weaker modes are not resolved because of the lower signal-to-noise ratio and spectral overlap with the graphene-related Raman response. Similarly, the powder spectrum exhibits reduced spectral quality because the irregular shape and size distribution of the HMTP crystallites make reproducible optical focusing difficult. Nevertheless, the comparison supports assigning the dominant peaks to HMTP vibrations, whose frequencies are not measurably affected by the different azimuthal domain orientations within our experimental resolution. The HMTP/SiC spectrum is therefore used in the main text for mode assignment because it provides the clearest molecular Raman fingerprint while avoiding the strong overlap between HMTP modes and graphene-related Raman features. The positions of the graphene G and 2D modes are indicated in Fig. S6 (c) by labeled arrows.

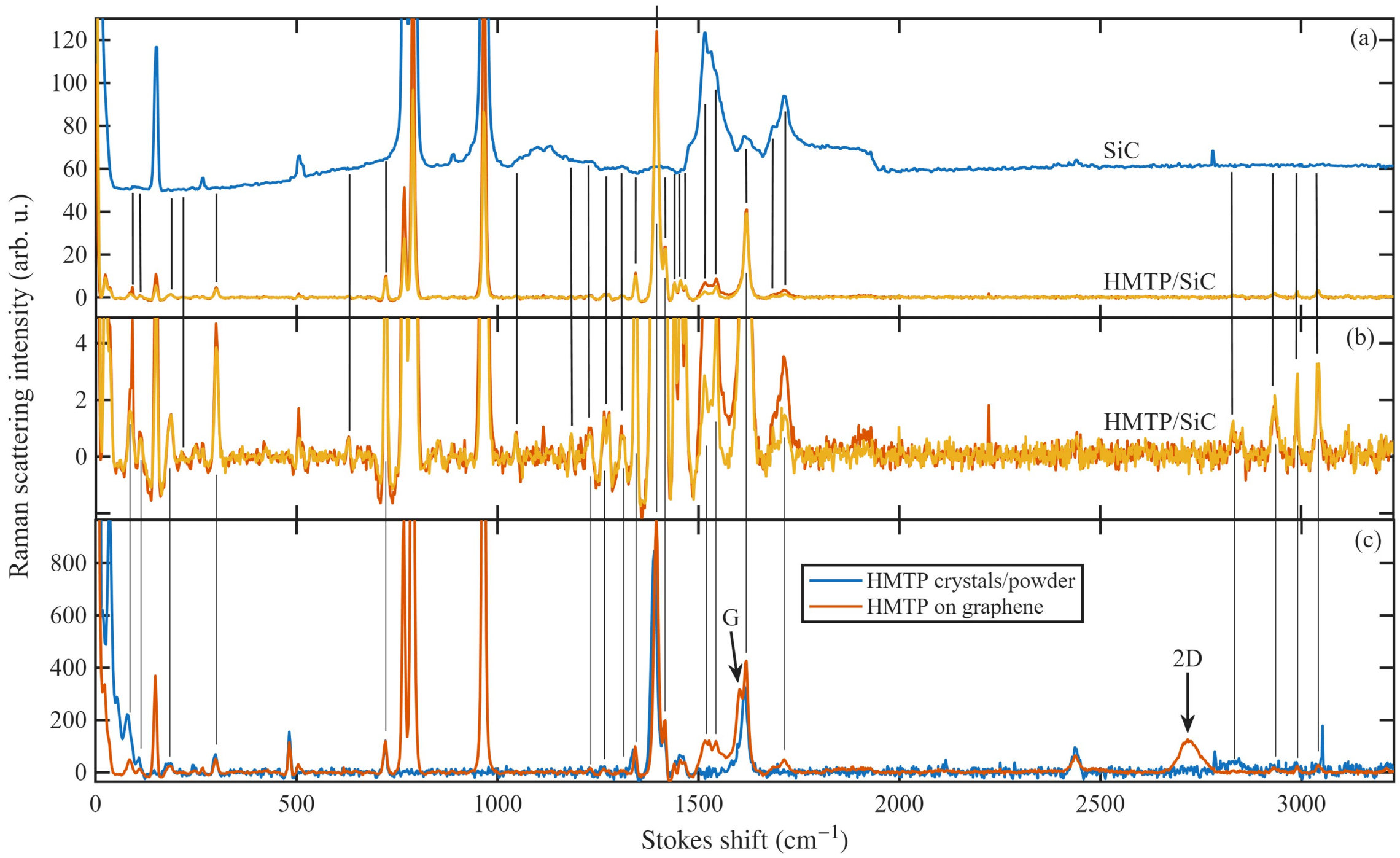

Figure S6: Raman comparison of HMTP on SiC, graphene, and in crystalline powder form. (a) Raman spectra of the bare SiC substrate and HMTP grown on SiC, corresponding to the data shown in the main text. (b) Magnified view of the low-intensity region from panel (a), emphasizing the characteristic HMTP vibrational modes. (c) Raman spectra of HMTP powder and HMTP grown on graphene.

7 Polarization-Resolved Photoluminescence

To cross-check the assignment of the major and minor photoluminescence peaks, we performed polarization-resolved PL measurements on the HMTP/graphene heterostructure. To minimize systematic artifacts from photobleaching, the analyzer angles were measured in a randomized order rather than sequentially, thereby avoiding a direct correlation between acquisition time and polarization angle. The laser spot size was $\approx 10\ \mu\text{m}$. The resulting angular dependence is summarized in Fig. S7. Without background subtraction, the integrated PL intensity is essentially isotropic within the experimental uncertainty [Fig. S7(a)]. This absence of a dominant macroscopic linear polarization is consistent with the high in-plane symmetry of the HMTP crystal structure. In particular, for the $P6_3/m$ structure with two inequivalent HMTP molecules in the unit cell, the transition dipoles are distributed over threefold/sixfold symmetry-equivalent molecular orientations, so that the ensemble-averaged PL response is expected to be nearly isotropic. After subtraction of the weak background contribution, a small residual angular modulation becomes visible [Fig. S7(b)]. The corresponding anisotropy is only on the order of 2–5%, which is much smaller than would be expected for a single-domain film with a strongly preferred transition-dipole orientation. We therefore do not assign this weak modulation to an intrinsic Davydov-branch polarization selection rule. Instead, it likely reflects small extrinsic symmetry-breaking effects, such as local strain, substrate anisotropy, finite domain statistics within the optical spot, or residual uncertainty associated with

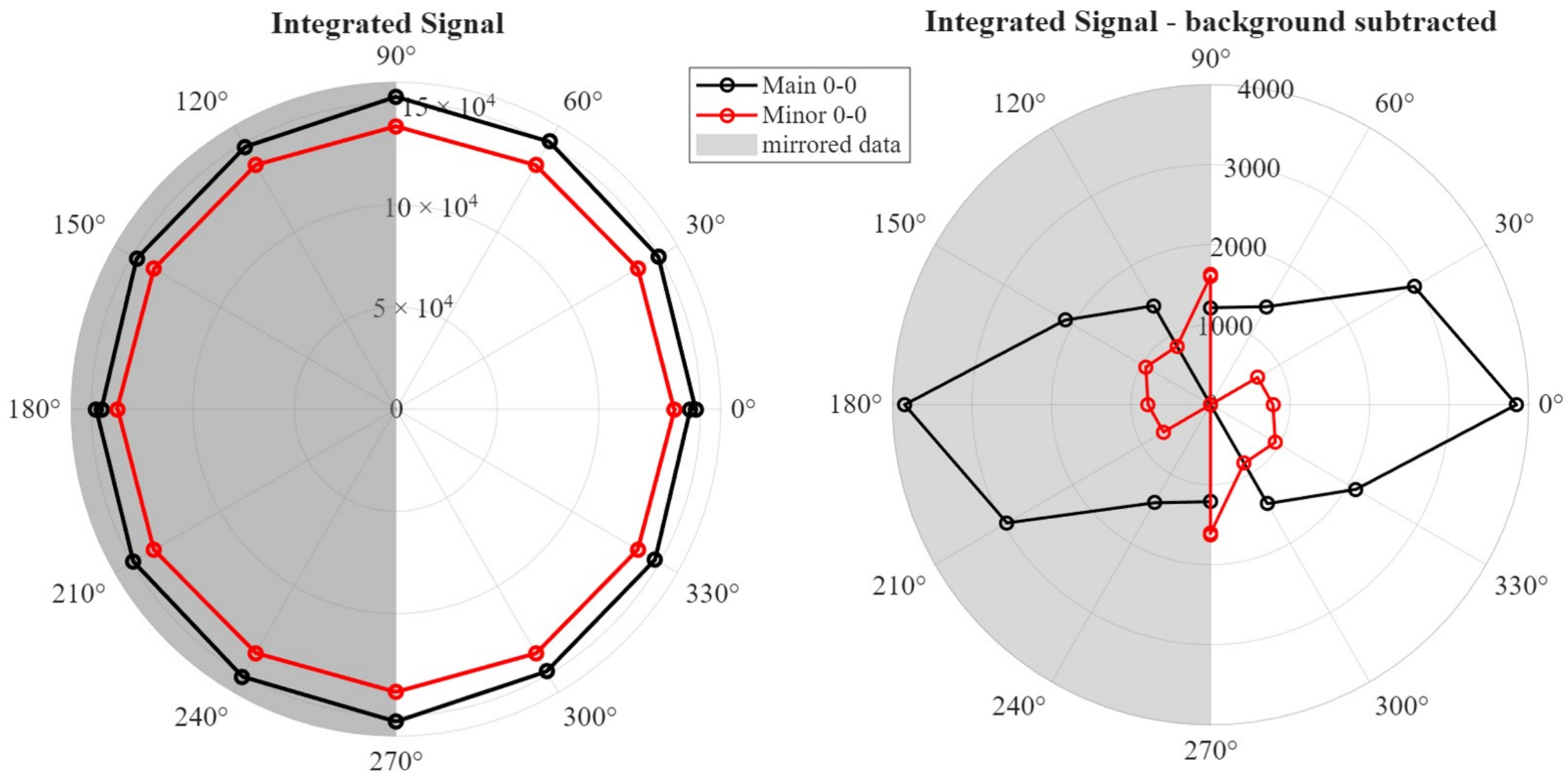

Figure S7: Polarization-resolved photoluminescence of the HMTP/graphene heterostructure. (a) Polar plot of the integrated PL intensity as a function of analyzer angle before background subtraction, showing an essentially isotropic response within the experimental uncertainty. (b) Polar plot after subtraction of the isotropic background contribution. A small residual anisotropy of approximately 2-5% is observed. The greyed sectors in the polar plots correspond to the angular range from $+90°$ to $+270°$, which was not measured independently. These data points were generated by copying the measured interval from $-90°$ to $+90°$ using the $180°$ periodicity of the linear-polarization measurement.

background subtraction. The polarization-resolved PL measurements are therefore consistent with an ensemble-averaged response of symmetry-related HMTP domains, including both rotational orientations with respect to graphene, rather than emission from a single macroscopically aligned transition dipole.

8 Tight-Binding Derivation of the HMTP Band Structure

To estimate the intermolecular coupling strength t from our ARPES data, we employ a nearest-neighbor tight-binding (TB) model based on the $P6_3/m$ crystalline symmetry of HMTP. The unit cell contains two inequivalent molecular sites, defining two sublattices ($\mathcal{A}$ and $\mathcal{B}$) as illustrated in Fig. S8.

8.1 Hopping Vectors and Coordination

We consider the eight primary hopping pathways for an exciton or charge carrier: six intra-sublattice neighbors within the ab-plane and two inter-sublattice neighbors along the c-axis stacking direction. For intra-sublattice neighbors ($\mathcal{A} \to \mathcal{A}$ or $\mathcal{B} \to \mathcal{B}$), each HMTP molecule has six

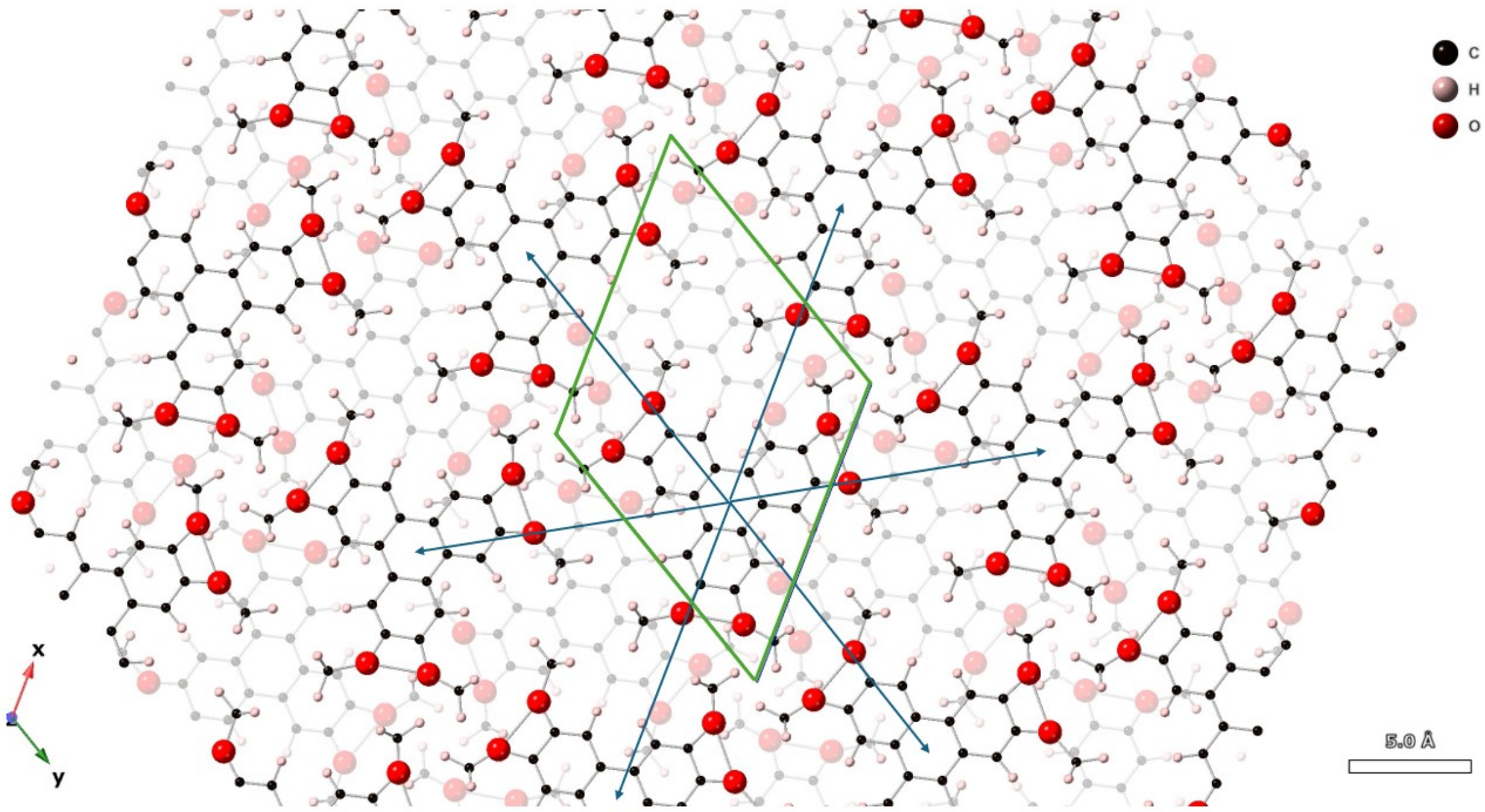

Figure S8: In-plane molecular packing and nearest-neighbor coupling. Top-down view of the HMTP crystal lattice highlighting the intra-sublattice arrangement. Two molecular layers are shown, with the underlying layer (sublattice $\mathcal{B}$) shaded in grey to distinguish it from the primary layer (sublattice $\mathcal{A}$). The hexagonal unit cell is indicated by the green rhombus. The six nearest-neighbor vectors within the ab-plane are denoted by blue arrows, representing the primary hopping pathways for the intra-sublattice interaction (t_{ab}). These vectors correspond to the six terms in the intra-sublattice sum $\sum_j e^{i\mathbf{k}\cdot\mathbf{R}_j}$ used in the tight-binding Hamiltonian. Atomic species are indicated by black (C), red (O), and white (H) spheres.

neighbors in the hexagonal plane at distance a:

$$\mathbf{R}_{1,2} = \pm a(1, 0, 0) \tag{S1}$$

$$\mathbf{R}_{3,4} = \pm a\left(-\frac{1}{2}, \frac{\sqrt{3}}{2}, 0\right) \tag{S2}$$

$$\mathbf{R}_{5,6} = \pm a\left(-\frac{1}{2}, -\frac{\sqrt{3}}{2}, 0\right) \tag{S3}$$

For inter-sublattice neighbors ($\mathcal{A} \to \mathcal{B}$), the two neighbors along the c-axis are at:

$$\mathbf{R}_{7,8} = \pm\left(0, 0, \frac{c}{2}\right). \tag{S4}$$

8.2 Tight-Binding Hamiltonian

The electronic states are described by the 2×2 Hamiltonian matrix in the basis of the two sublattices:

$$\mathcal{H}(\mathbf{k}) = \begin{pmatrix} H_{AA}(\mathbf{k}) & H_{AB}(\mathbf{k}) \\ H_{BA}(\mathbf{k}) & H_{BB}(\mathbf{k}) \end{pmatrix} \tag{S5}$$

The diagonal terms, representing the intra-sublattice dispersion, are obtained by summing the phase factors over the six in-plane neighbors:

$$H_{AA}(\mathbf{k}) = \epsilon_0 - 2t_{ab}\left[\cos(k_x a) + 2\cos\left(\frac{k_x a}{2}\right)\cos\left(\frac{\sqrt{3}k_y a}{2}\right)\right] \tag{S6}$$

with

$$H_{AA}(\mathbf{k}) = H_{BB}(\mathbf{k}). \tag{S7}$$

The off-diagonal term represents the coupling between the two sublattices along the stacking direction:

$$H_{AB}(\mathbf{k}) = H^*_{BA}(\mathbf{k}) = -2t_z\cos\left(\frac{k_z c}{2}\right) \tag{S8}$$

8.3 Dispersion and Bandwidth

Diagonalizing $\mathcal{H}(\mathbf{k})$ yields the energy eigenvalues

$$E_\pm(\mathbf{k}) = H_{AA}(\mathbf{k}) \pm |H_{AB}(\mathbf{k})|. \tag{S9}$$

The total bandwidth (BW) is defined by the difference between the global maximum and minimum of the energy bands. For a simplified isotropic case where the hopping integrals are comparable ($t_{ab} \approx t_z = t$), the extrema occur at the Γ point (E_{min}) and the M or K points (E_{max}). At the Γ point ($\mathbf{k} = 0$):

$$H_{AA}(0) = \epsilon_0 - 6t, \quad H_{AB}(0) = -2t \implies E_{min} = \epsilon_0 - 8t.$$

At the zone boundary (namely at the K point where $H_{AA} = \epsilon_0 + 3t$ and k_z allows $H_{AB} = +2t$):

$$E_{max} = \epsilon_0 + 5t \tag{S10}$$

Thus, the total theoretical bandwidth is:

$$BW = E_{max} - E_{min} = 13t \tag{S11}$$

This linear relationship allows us to directly map the ≈ 40 meV dispersion observed in ARPES to an intermolecular coupling strength $t \approx 3$ meV, confirming the heavy-exciton, strong-coupling regime.

9 Vibronic Transitions and Herzberg-Teller Corrections

In this section, we provide a formal derivation of the vibronic intensity profiles based on the Franck-Condon (FC) principle and the first-order Herzberg-Teller (HT) correction. We model the system using a displaced harmonic oscillator (DHO) manifold, where the ground and excited states are represented by linear harmonic oscillators (LHO) with a relative displacement in the generalized nuclear coordinate Q.

9.1 Displaced Harmonic Oscillator

The generalized coordinate Q for a phonon mode with frequency ω and effective mass M_{eff} is expressed in terms of the bosonic creation ($a^\dagger$) and annihilation (a) operators:

$$Q = \sqrt{\frac{\hbar}{2M_{\text{eff}}\omega}}(a + a^\dagger). \tag{S12}$$

The displacement of the excited state potential energy surface relative to the ground state is given by $\Delta Q = \sqrt{2\hbar S/M_{\text{eff}}\omega}$, where S is the dimensionless Huang-Rhys factor. We utilize the displacement operator $D(\sqrt{S})$[S7] to map the ground state wavefunction onto the excited state manifold:

$$D = e^{\sqrt{S}(a^\dagger - a)} = e^{-S/2} e^{\sqrt{S}a^\dagger} e^{-\sqrt{S}a}, \tag{S13}$$

where the second equality follows from the Baker-Campbell-Hausdorff identity. The operator D acts on the operators as $D^\dagger a D = a + \sqrt{S}$ and $D^\dagger a^\dagger D = a^\dagger + \sqrt{S}$, shifting the expectation value of the position:

$$\langle S|Q|S\rangle = \langle 0|D^\dagger Q D|0\rangle = \sqrt{\frac{2\hbar S}{M_{\text{eff}}\omega}}. \tag{S14}$$

9.2 Franck-Condon Intensities

The transition amplitude in the Franck-Condon approximation assumes a constant electronic transition dipole moment μ_0. The intensity $I_{0\to n}$ of the n-th vibronic replica is proportional to the square of the overlap integral (Franck-Condon factor):

$$D|0\rangle = e^{-S/2} \sum_{k=0}^{\infty} \frac{S^{k/2}}{\sqrt{k!}} |k\rangle. \tag{S15}$$

The overlap $\langle n|D|0\rangle$ yields the well-known Poissonian distribution for intensity:

$$I_{0\to n} \propto |\mu_0 \langle n|D|0\rangle|^2 \propto e^{-S} \frac{S^n}{n!}. \tag{S16}$$

9.3 Herzberg-Teller Correction

To account for the coordinate dependence of the transition dipole moment, we expand $\mu(Q)$ to the first order[S8]:

$$\mu(Q) = \mu_0 + \mu' Q, \tag{S17}$$

where $\mu' = (\partial\mu/\partial Q)_{Q_0}$. The total transition matrix element M becomes:

$$M = \mu_0 \langle n|D|0\rangle + \mu' \langle n|QD|0\rangle. \tag{S18}$$

Using Eq. (S12), the second term is evaluated as:

$$\langle n|QD|0\rangle = \sqrt{\frac{\hbar}{2M_{\text{eff}}\omega}} \left(\langle n|Da^\dagger|0\rangle + 2\sqrt{S}\langle n|D|0\rangle \right). \tag{S19}$$

Following the bosonic algebra for $\langle n|D|1\rangle$, we first identify how the displacement operator acts on the state $|1\rangle$.

$$\begin{aligned} D\,|1\rangle &= e^{-\frac{S}{2}} e^{\sqrt{S}a^{+}} e^{-\sqrt{S}a}\,|1\rangle = \\ &= e^{-\frac{S}{2}} e^{\sqrt{S}a^{+}} \sum_{k=0}^{\infty} \frac{(-\sqrt{S}a)^k}{k!}\,|1\rangle = \\ &= e^{-\frac{S}{2}} e^{\sqrt{S}a^{+}} (|1\rangle - \sqrt{S}\,|0\rangle) \end{aligned} \tag{S20}$$

We used the observation that in the sum; only the first two terms, $k = \{0, 1\}$, are non-zero. We can apply the second part of the displacement operator, containing the creation operator

$$\begin{aligned} D\,|1\rangle &= e^{-\frac{S}{2}} e^{\sqrt{S}a^{+}} (|1\rangle - \sqrt{S}\,|0\rangle) = \\ &= e^{-\frac{S}{2}} \sum_{k=0}^{\infty} \frac{(\sqrt{S}a^{+})^k}{k!} (|1\rangle - \sqrt{S}\,|0\rangle). \end{aligned} \tag{S21}$$

Using $(a^{+})^k\,|0\rangle = \sqrt{k!}\,|k\rangle$, and $(a^{+})^k\,|1\rangle = \sqrt{(k+1)!}\,|k+1\rangle$, we have

$$\begin{aligned} &D\,|1\rangle = \\ &\quad e^{-\frac{S}{2}} \sum_{k=0}^{\infty} \left[\frac{S^{k/2}}{k!} \sqrt{(k+1)!}\,|k+1\rangle - \sqrt{S} \frac{S^{k/2}}{k!} \sqrt{k!}\,|k\rangle \right]. \end{aligned} \tag{S22}$$

Noticing that the second term equals $-\sqrt{S}D\,|0\rangle$, we get

$$\langle n|\,D\,|1\rangle = \langle n| \left[e^{-\frac{S}{2}} \sum_{k=0}^{\infty} \frac{S^{k/2}}{k!} \sqrt{(k+1)!}\,|k+1\rangle - \sqrt{S}D\,|0\rangle \right]. \tag{S23}$$

Using the orthonormality of the LHO eigenstates, we have

$$\langle n|\,D\,|1\rangle = e^{-\frac{S}{2}} \frac{S^{(n-1)/2}}{(n-1)!} \sqrt{n!} - \sqrt{S}\,\langle n|\,D\,|0\rangle\,. \tag{S24}$$

Using Eq. (S15), the first term is $\frac{n}{\sqrt{S}}\,\langle n|\,D\,|0\rangle$, and we get the final result of the matrix element

$$\langle n|\,D\,|1\rangle = \left(\frac{n}{\sqrt{S}} - \sqrt{S} \right) \langle n|\,D\,|0\rangle\,. \tag{S25}$$

Combining the terms with Eq. (S19), we arrive at:

$$M = \mu_0 \langle n|D|0\rangle \left[1 + \frac{\mu'}{\mu_0} \sqrt{\frac{\hbar}{2M_{\text{eff}}\omega}} \left(\frac{n+S}{\sqrt{S}} \right) \right]. \tag{S26}$$

Defining the dimensionless Herzberg-Teller coupling constant as $\alpha = \frac{\mu'}{\mu_0}\sqrt{\frac{\hbar}{2M_{\text{eff}}\omega}}$, the final expression for the vibronic emission intensity is:

$$I_{0\to n}^{\text{em}} \propto \frac{S^n}{n!}\left(1+\alpha\frac{n+S}{\sqrt{S}}\right)^2. \tag{S27}$$

While the emission profile is governed by the matrix element $\langle n|QD|0\rangle$, the absorption process requires the evaluation of the transition amplitude starting from the ground state and projecting onto the n-th vibrational level of the excited state manifold. This is represented by:

$$M_{\text{abs}} = \langle Dn|(\mu_0+\mu' Q)|0\rangle = \mu_0\langle n|D^\dagger|0\rangle + \mu'\langle n|D^\dagger Q|0\rangle. \tag{S28}$$

In this case, the operator order in the second term is $D^\dagger Q$. Unlike the emission derivation in Eq. (S19), where the commutator $[Q, D]$ introduced a $2\sqrt{S}$ term, the absorption matrix element depends directly on the action of the coordinate operator on the ground state. The vibronic correction then yields a factor proportional to $(n/\sqrt{S}-\sqrt{S})$. Consequently, the intensity distribution for absorption, including the sign change of α that stems from the $D^\dagger$ operator [$D^\dagger(\sqrt{S}) = D(-\sqrt{S})$], is given by:

$$I_{0\to n}^{\text{abs}} \propto \frac{S^n}{n!}\left(1+\alpha\frac{n-S}{\sqrt{S}}\right)^2. \tag{S29}$$

This formulation accounts for the non-Condon effects observed in our experimental absorption spectra, where α parameterizes the vibronic borrowing strength relative to the purely electronic transition.

Supplementary References

[S1] Shetty, N. *et al.* Scalable fabrication of edge contacts to 2d materials: Implications for quantum resistance metrology and 2d electronics. *ACS Applied Nano Materials* **6**, 6292–6298 (2023).

[S2] Berger, C. *et al.* Ultrathin epitaxial graphite: 2d electron gas properties and a route toward graphene-based nanoelectronics. *Journal of Physical Chemistry B* **108**, 19912–19916 (2004).

[S3] Emtsev, K. V. *et al.* Towards wafer-size graphene layers by atmospheric pressure graphitization of silicon carbide. *Nature Materials* **8**, 203–207 (2009).

[S4] Kunc, J. *et al.* Effect of residual gas composition on epitaxial growth of graphene on sic. *Physical Review Applied* **8**, 044011 (2017).

[S5] Shestopalov, M., Stará, V., Rejhon, M. & Kunc, J. Annealing, design and long-term operation of graphite crucibles for the growth of epitaxial graphene on sic. *Journal of Crystal Growth* **651**, 127988 (2025).

[S6] Nečas, D. & Klapetek, P. Gwyddion: an open-source software for spm data analysis. *Central European Journal of Physics* **10**, 181–188 (2012).

[S7] Feynman, R. P. An operator calculus having applications in quantum electrodynamics. *Physical Review* **84**, 108–128 (1951).

[S8] Kundu, S., Roy, P. P., Fleming, G. R. & Makri, N. Franck-condon and herzberg-teller signatures in molecular absorption and emission spectra. *Journal of Physical Chemistry B* **126**, 2899–2911 (2022).
